# Clinical Features and Outcomes of Bilateral Atypical Femoral Fractures

**DOI:** 10.1155/2020/8824756

**Published:** 2020-07-23

**Authors:** Kosuke Hamahashi, Toshihiro Noguchi, Yoshiyasu Uchiyama, Masato Sato, Masahiko Watanabe

**Affiliations:** Department of Orthopaedic Surgery, Surgical Science, Tokai University School of Medicine, 143 Shimokasuya, Isehara, Kanagawa 259-1193, Japan

## Abstract

Bilateral atypical femoral fractures (AFFs) are relatively rare. In this report, we retrospectively researched clinical features and outcomes of bilateral AFFs treated at our institution. We previously treated 4 patients (8 limbs) with intramedullary nailing for complete AFFs (6 limbs) and incomplete AFFs (2 limbs). The mean age at the first operation was 53.3 years, and all patients were female. Of the 4 patients, two had breast cancer, and another two had systemic lupus erythematosus. Three of them received bisphosphonates, and 2 received denosumab, proton pump inhibitor, or glucocorticoid therapy. Only 2 of 6 cases of incomplete AFFs had prodromal pain before progressing to complete fracture. The mean interval from the first surgery to contralateral fracture or prophylactic surgery was 16 months. Radiographically, complete bone union was achieved in 6 limbs. However, a small gap at the lateral cortex of fracture site remained in 2 limbs. Finally, all of the patients were pain-free and able to walk without a cane. It is absolutely necessary to confirm contralateral femoral conditions; however, prediction of progression to complete fracture based solely on prodromal pain was difficult. Therefore, we should advise patients about the danger of progression to complete AFFs even if they are asymptomatic, and a prophylactic surgery should be performed after obtaining informed consent.

## 1. Introduction

According to the Japanese Orthopaedic Association registry report, there were 344 cases of AFFs in 2015, 448 in 2016, and 505 in 2017. Among them, bilaterally affected cases were 37 (10.8%), 69 (15.4%), and 61 (12.1%), respectively [1]. In a study involving 142 participants, Dell et al. reported that contralateral fractures occurred in 32 patients (22.5%) of the AFF group and were located at the same anatomic location on the contralateral side as on the ipsilateral side [2]. In another study involving 39 participants, Meier et al. reported that contralateral fractures occurred in 11 patients (28.2%) in the AFF group. They also mentioned that the contralateral fracture was of the same type as the first fracture in all cases [3]. Saita et al. reported that the site of the AFFs along the femoral diaphysis was highly correlated with the deviation between the anatomical axis of the femur and tibia and the mechanical axis of the lower limb [4]. Lateral femoral bowing and varus hip geometry, which increase loading forces on the lateral femoral cortex, may increase AFF risk [5]. In a Japanese population, patients who developed AFFs had significantly greater curvature of the femoral diaphysis than age- and gender-matched controls [6]. However, this study describes 4 cases of bilateral AFFs without curvature of the femvoral diaphysis. We retrospectively studied the clinical features and outcomes of bilateral AFFs treated in our institution.

## 2. Case Presentation

This study was approved by the Institutional Review Board for Clinical Research of our University (no. 19R301; approval date; March 12, 2020). The patients and/or their families were informed that data from the cases would be submitted for publication, and they provided written informed consent.

We previously treated 4 Japanese female patients (8 limbs) between 2014 and 2018 in our institution. All patients underwent intramedullary nailing for both complete AFFs (6 limbs) and incomplete AFFs (2 limbs). In all cases, treatment began when the patient had complete ipsilateral AFFs. At that time, periosteal or endosteal thickening of the lateral cortices (“beaking” or “flaring”), or incomplete fractures were already present at the contralateral femur. The mean age at the first operation was 53.3 years (range 41-58). Mean height, weight, and body mass index were 151.5 cm (range 149-154), 47 kg (range 42-52), and 20.5 kg/m^2^ (range 18.5-22), respectively. There were no obese patients. The mean follow-up period was 24 months. Radiographically, complete bone union was achieved in 6 limbs. However, a small gap at the lateral cortex of fracture site remained in 2 limbs. Finally, all of the patients were pain-free and able to walk without a cane. The medication history and clinical features of each patient are summarized in Tables 1 and 2.

### 2.1. Case 1

**Figure 1:**
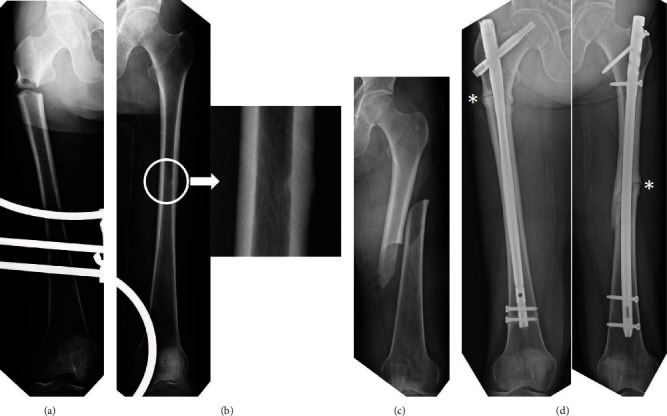
(a) The patient suffered a subtrochanteric AFF without prodromal pain on the right side as a result of a fall. (b) At that time, the left femoral roentgenogram showed periosteal and endosteal thickening of the lateral cortex at the diaphysis (circle; see magnification). (c) Fifteen months later, the patient suffered a diaphyseal AFF on the left side by falling without prodromal pain. (d) A small gap at the lateral cortex of fracture site (asterisk) remained on the right and left sides at 29 and 14 months after the surgery, respectively.

A 58-year-old female patient, who had received denosumab therapy for osteoporosis for more than 3 years, suffered a subtrochanteric AFF on the right side by falling from a standing height without prodromal pain (Figure 1(a)). She underwent an internal fixation with intramedullary nailing. At that time, the left femoral roentgenogram showed periosteal and endosteal thickening of lateral cortex at the diaphysis (Figure 1(b)). Unfortunately, this sign was missed, and denosumab therapy was continued. Fifteen months later, she suffered a diaphyseal AFF on the left side by falling from a standing height without prodromal pain (Figure 1(c)). She underwent an internal fixation with intramedullary nailing. A small gap at the lateral cortex of the fracture site remained on the right and left sides at 29 and 14 months after surgery, respectively (Figure 1(d)).

### 2.2. Case 2

**Figure 2:**
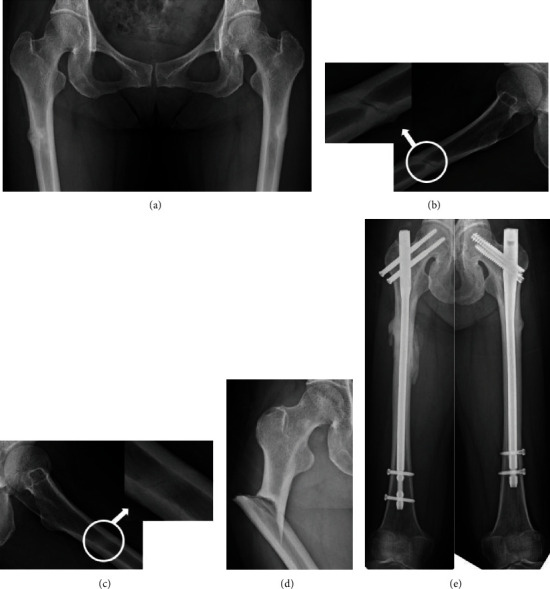
(a) The patient experienced bilateral thigh pain, and incomplete AFFs were detected. (b, c) Incomplete fracture lines, so called “dreaded black lines,” were clearly present on lateral view (circles; see magnifications). (d) The patient suffered a subtrochanteric AFF on the right side by falling. (e) Complete bone unions were achieved on the right and left sides at 24 and 15 months after surgery, respectively.

A 57-year-old female patient had received denosumab for more than 2 years, which followed BP (zoledronic acid hydrate) therapy for metastatic vertebral tumor of breast cancer for more than 10 years. She had experienced bilateral thigh pain, and incomplete AFFs were detected (Figures 2(a)–2(c)). She was advised about the risk of progression to complete AFFs, yet she refused surgery. Three months later, she suffered a subtrochanteric AFF on the right side by falling from a standing height (Figure 2(d)) and underwent an internal fixation with intramedullary nailing. Nine months later, she underwent prophylactic internal fixation due to persisting thigh pain on the left side. Complete bone unions were achieved on the right and left sides at 24 and 15 months after surgery, respectively (Figure 2(e)).

### 2.3. Case 3

**Figure 3:**
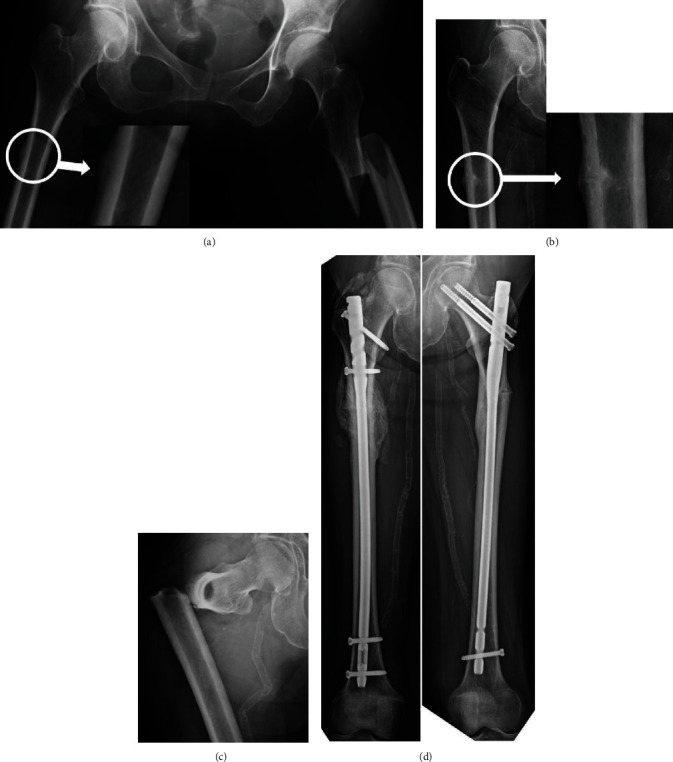
(a) The patient suffered a subtrochanteric AFF on the left side by sprain. At that time, the right femoral roentgenogram showed periosteal thickening of the lateral cortex at the subtrochanter (circle; see magnification). (b) Periosteal thickening of the lateral cortex progressed to incomplete fracture (circle; see magnification). (c) Incomplete fracture progressed to complete fracture by falling. (d) Complete bone unions were achieved on the left and right sides at 58 and 25 months after the surgery, respectively.

A 57-year-old female patient, who had received BP therapy for osteoporosis and prednisolone (10 mg/day) for systemic lupus erythematosus for no less than 10 years, was injured with subtrochanteric AFF on the left side by spraining her leg without prodromal pain. She underwent an internal fixation with intramedullary nailing. At that time, the right femoral roentgenogram showed periosteal thickening of the lateral cortex at subtrochanter (Figure 3(a)). Two years and 9 months after surgery, she experienced thigh pain on the right side, and the roentgenogram showed an incomplete fracture (Figure 3(b)). The patient was indecisive about undergoing surgery. In the meantime, the incomplete fracture progressed to a complete fracture when she experienced a fall from a standing height (Figure 3(c)). She underwent an internal fixation with intramedullary nailing. Complete bone unions were achieved on the left and right sides at 58 and 25 months after surgery, respectively (Figure 3(d)).

### 2.4. Case 4

**Figure 4:**
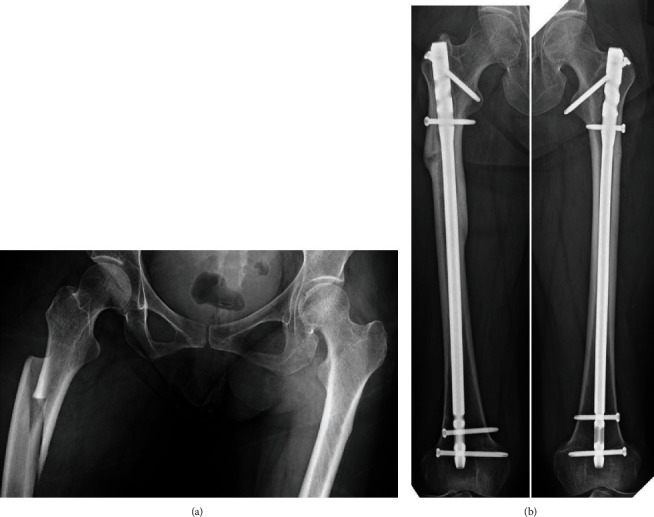
Both femur roentgenograms of case 4 at the time of injury (a) and final follow-up (b). There were no curvatures at both femoral diaphyses.

A 41-year-old female patient, who had received BP therapy for osteoporosis since 3 years and prednisolone (8 mg/day) for systemic lupus erythematosus, was injured with subtrochanteric AFF on the right side by falling from a standing height without prodromal pain (Figure 4(a)). She underwent an internal fixation with intramedullary nailing. At that time, the left femoral roentgenogram showed periosteal thickening of the lateral cortex at the subtrochanter. Seven months later, she decided to undergo prophylactic internal fixation based on our strong recommendation. Complete bone union was achieved on the right side at 19 months after surgery (Figure 4(b)).

## 3. Discussion

AFFs are primarily associated with prolonged use of BPs but have also been reported in patients who do not use BP [7]. Also, patients who receive denosumab for osteoporosis and metastatic bone disease are susceptible to AFFs [8, 9]. Furthermore, patients with autoimmune diseases and using glucocorticoids develop risk factors for not only osteoporotic fractures but also AFFs [10]. Zenke et al. reported that AFF could be primarily caused by 3 factors: long-term use of BPs, drugs other than BPs for comorbidity treatment, and femoral lateral bowing [11]. In this study, every case had multiple risk factors associated with medical conditions and medication history. However, there was no case that had curvature of the femoral diaphysis, as shown in Figures 1–4. Probyn et al. reported a strong correlation between bilateral AFF locations, with 58 of 76 (76.3%) occurring within <5 cm and 41 of 76 (53.9%) within ≤2.5 cm. They concluded that bilateral AFFs commonly have similar imaging features, including location along the femur [12]. In this study, the patient described in Case 1 first experienced her right femoral “subtrochanteric” fracture and later the left femoral “diaphyseal” fracture; this seems a relatively rare case.

When ipsilateral AFF occurred, it was necessary to check a roentgenogram of the contralateral femur. We should have considered prophylactic surgery when incomplete AFF was detected. However, there was no consensus among surgeons whether prophylactic surgery should be performed or not. Koh et al. reported symptomatic cortical stress reactions; in the presence of the “dreaded black line,” known as a radiologic feature indicative of stress fracture nonunion [13] (Figures 2(b) and 2(c)), these present an increased risk for complete stress fractures. They recommended prophylactic surgery under these circumstances [14]. Toro et al. mentioned that in patients with asymptomatic incomplete fractures associated with simultaneous contralateral complete fracture, prophylactic surgery is the gold standard for early weight bearing; however, the ultimate decision depends on patient preferences. Furthermore, prophylactic surgery is the treatment of choice in patients who are at high risk for progression of the AFF, namely, those who are on long-term antiresorptive therapy and/or are highly compliant with this treatment, proton pump inhibitor, or glucocorticoid users and patients who have sustained a contralateral AFF [15]. Banffy et al. reported that patients who underwent prophylactic surgery for incomplete AFFs had shorter hospital stays than those who had surgery after complete AFFs [16]. Furthermore, several studies have demonstrated that complete AFFs have higher rates of perioperative complications [16, 17]. In this study, only 2 out of 6 incomplete AFFs had prodromal pain before progressing to complete fractures. It was suggested that diagnosis of the progression to complete fracture was difficult based solely on prodromal pain, as it might not be a useful predictor for bilateral cases.

In conclusion, we treated 4 cases (8 limbs) with intramedullary nailing for both complete AFFs (6 limbs) and incvomplete AFFs (2 limbs). All cases had multiple risk factors for AFFs; however, there were no cases with curvature of the femoral diaphysis. It was suggested that prediction of the progression to complete fracture was difficult based solely on prodromal pain. Therefore, we should advise patients about the danger of progression from incomplete to complete AFFs, even if they are asymptomatic, and prophylactic surgery should be recommended for bilateral AFFs.

## Figures and Tables

**Table 1:** Medication history of each patient.

	Case 1	Case 2	Case 3	Case 4
Bisphosphonate				
Zoledronic acid hydrate		○		
Alendronate sodium hydrate			○	
Minodronic acid hydrate				○
Denosumab	○	○		
Proton pump inhibitor				
Esomeprazole magnesium hydrate	○			
Lansoprazole		○		
Glucocorticoid (prednisolone)			10 mg/day	8 mg/day

**Table 2:** Clinical features of each patient.

	Case 1	Case 2	Case 3	Case 4
Age (years)	58	57	57	41
Height (cm)	151	154	149	152
Weight (kg)	42	52	43	51
Body mass index (kg/m^2^)	18.5	22	19.3	22
Fracture location				
Right	Subtrochanter	Subtrochanter	Subtrochanter	Subtrochanter
Left	Diaphysis	Subtrochanter	Subtrochanter	Subtrochanter
Fracture type				
Right	Complete	Complete	Complete	Complete
Left	Complete	Incomplete	Complete	Incomplete
Bone union				
Right	Gap+	Complete	Complete	Complete
Left	Gap+	Complete	Complete	Complete

## Data Availability

The datasets used and/or analyzed during the current study are available from the corresponding author on reasonable request.
